# Association between hair dye use and cancer in women: a systematic review and meta-analysis of case-control studies

**DOI:** 10.4314/ahs.v22i2.36

**Published:** 2022-06

**Authors:** Mohadeseh Ahmadi, Majid Saeedi, Akbar Hedayatizadeh-Orman, Mahboobeh Eslami, Ghasem Janbabai, Reza Alizadeh-Navaei

**Affiliations:** 1 Gastrointestinal Cancer Research Center, Non-Communicable Diseases Institute, Mazandaran University of Medical Sciences, Sari, Iran; 2 Pharmaceutical Sciences Research Center, Haemoglobinopathy Institute, Mazandaran University of Medical Sciences, Sari, Iran; 3 Department of Internal Medicine, School of Medicine, Shariati Hospital, Tehran University of Medical Sciences, Tehran, Iran

**Keywords:** Hair dye, cancer, meta-analysis

## Abstract

**Background:**

The use of hair dye for cosmetic purposes appears to be increasing worldwide. As 50–80% of women use hair dye throughout their lifetimes, the possible association between hair dye use and cancer is a public health concern.

**Method:**

This systematic review was performed by retrieving studies from PubMed, Scopus, WOS, and ProQuest databases. The inclusion criteria were case-control studies evaluating the association between hair dye use and cancer in women. Women with cancer who have used any hair dye were the focus of our study.

**Results:**

The present study combined 28 studies, to assess the association between hair dye use and cancer. The pooled odds ratio (OR) of hematopoietic system cancers among those who have generally ever used any type of hair dyes was 1.10 (95% CI:1.01–1.20) in 17 studies. In 11 studies investigating hair dye made before and after 1980 as a risk factor for cancer, the pooled OR for cancer was 1.31(95% CI:1.08–1.59). Likewise, in the 13 studies that evaluated the association of light and dark hair dye with cancer, the risk among those using dark hair dye increased by 9%, compared to non-users (OR=1.09; 95% CI:0.95–1.25).

**Conclusion:**

The present study suggests that, although the use of hair dye may increase the risk of cancer among users, a more detailed evaluation is required to assess the type of hair dye use in terms of guidelines and metrics.

## Introduction

Hair dye is a commercial product that numerous people, particularly women, use to change their appearance, including covering their grey hair^1^. Hair dye products may be classified as permanent, semi-permanent and, temporary; permanent hair dyes are oxidative, whereas semi-permanent and temporary dyes are non-oxidative 2. These products differ in chemical formulation regarding the frequency of use^3^ and penetration of the hair shaft. Permanent dyes account for ∼80% of hair dye sales contain colorless intermediates derived from phenylalanine and provide the desired. Dyes produced during the oxidation reaction penetrate the hair shaft and cause oxidative damage^4^–^6^. Therefore, it may be possible to attribute some of the damage caused by hair dye to the coloring process, apart from its gradients. In addition, dark colors include a higher concentration of intermediates and precursors that react in hydrogen peroxide to form pigment molecules. Non-oxidative, temporary, and semi-permanent dyes contain compounds that directly color the hair^7^, ^8^. One of the most important facts is that compounds such as phenylenediamine and para-phenylenediamine (PPD), which are possible carcinogens, can cause cytogenetic changes and damage to the DNA^9^, ^10^.

Regarding the controversial statements of available related studies and the uncertainty on the risk of cancer from using hair dyes, a systematic review and meta-analysis were conducted to investigate the various reports on the effects of hair dye exposure. Various types of cancer, the effects of the color intensity, and other significant factors were investigated.

## Materials and methods

Protocol. The protocol was submitted to Prospero under the registration code CRD42017074393. This systematic review was performed based on PRISMA guidelines^11^.

Eligibility criteria. All case-control studies, including population-based and hospital-based case-control studies, were selected. Based on the primary outcome, only studies that evaluated any cancer were included, whereas other outcomes, such as any Medical Syndromes and Cardiovascular diseases, were not considered. Other studies, such as case series and case reports, were excluded. Studies in men were excluded, and when studies included both men and women participants, only data from women were extracted for meta-analysis. Furthermore, one article published in the other language was excluded.

Information source. A search was conducted through four databases: PubMed, Scopus, ProQuest, and Web of Science, from January 1990 through 30 July 2017. The search strategy included the keywords ‘cancer’ AND ‘hair dye’. Two study investigators performed the search independently (MA and ME) and were checked by a third investigator (RAN).

Search strategy. These search terms were used to perform a final search through the PubMed database as follows:

(‘hair dye’[tiab] OR (dyes[tiab] AND hair[tiab]) OR ‘hair colorants’[tiab] OR (colorants[tiab] AND hair[tiab]) OR (‘coloring agents’[tiab] AND hair[tiab]) OR (agents[tiab] AND ‘hair coloring’[tiab]) OR ‘hair coloring agents’[tiab]) AND (cancer[tiab] OR neoplasia[tiab] OR neoplasias[tiab] OR neoplasm[tiab] OR tumor[tiab] OR ‘benign neoplasm’[tiab] OR (neoplasm[tiab] AND benign[tiab]) OR malignancy[tiab] OR cancer[tiab]) AND (1990/01/01:2017/7/31[dp]).

### Study selection

This phase was performed in 3 steps. First, duplicate publications were eliminated. Subsequently, each study was screened independently for eligibility criteria (ME and MA); disagreements were resolved by a subject expert (RAN). Finally, the studies screened in the previous steps were selected based on full-text assessment independently by two reviewers (RAN and AHO).

### Risk of bias assessment

To evaluate the methodological quality of the selected studies, the Newcastle-Ottawa Scale (NOS) tool was used^12^. Using this tool, the quality of the studies was evaluated according to three sections [selection with 4 scores for 4 items, comparability with 3 scores for two things (since one item may have 2 scores), and outcome with 3 scores for 3 items]. This process was conducted by two researchers (RAN and MA). Our cut-off was estimated based on the included studies and subject expert opinion as follows: 1–3, poor; 4–6, moderate; and 7–9, good.

### Data collection process

Extraction of data was performed by two reviewers independently (RAN and MA), while >90% of the items were in agreement and other items were resolved by consensus. The extraction data form included general information, quality assessment score, population description, and other related variables. The statistical section of this form categorized OR according to the type of use, type of color, duration of use, and use before/after 1980.

### Statistical analysis

Data were analyzed by the STATA 11 software (StataCorp LP). Heterogeneity was assessed by the I2 index and Q test. The random-effects model was used based on the heterogeneity index for pooled estimation, and funnel and forest plots were drawn. A value of 0% indicates no observed heterogeneity, and larger values show increasing heterogeneity.

## Results

### Study selection

By searching the databases, 1,201 articles were initially obtained; after removing duplicate publications, 428 articles remained.

### Study characteristics

**Fig 1:**
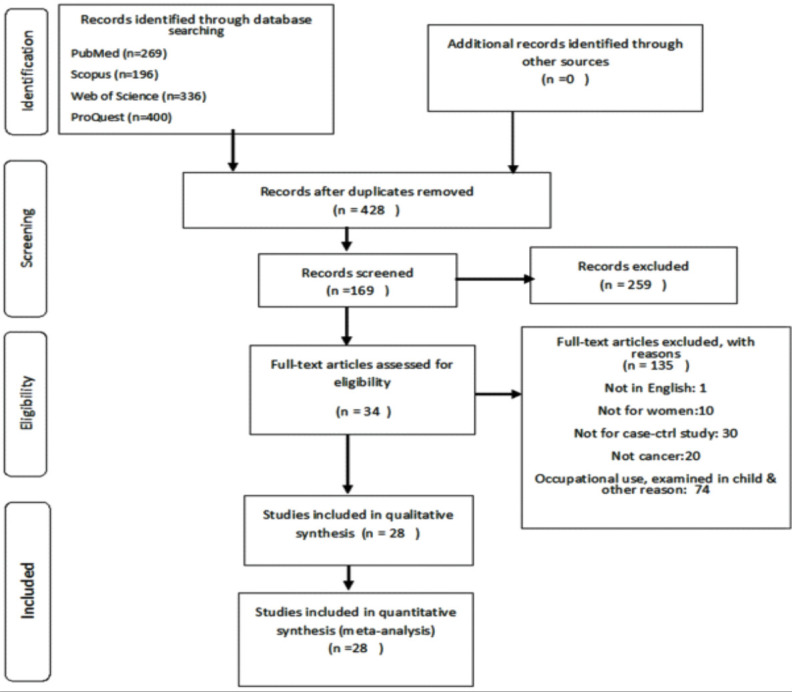
Overview of study selection according to the PRISMA statement

A total of 166 articles were identified after the screening of the title and abstract. Finally, 32 articles were selected following a full-text review. The study selection process is presented as the PRISMA flowchart in Fig.1. A Summary of the characteristics of each included study is presented in Table I 13–40.

**Table I:** Summary of characteristics of included studies

First author, year (Refs.)	Country	Race	Occupation	Design	N	Controls		Cases	
						Mean age, years	N	Mean age, years	QAS
Koenig, 1990 13	USA	White	ND	Case-control	609	56	294	60	7
Zahm,1991 14	USA	ND	ND	Population-based case-control	322	ND	NHL, n=106; Hodgkin, n=16; MM, n=24; leukemia, n=9	ND	8
Herrinton,1994 15	USA	White, others	Related	Population-based case-control	267	NR	114	NR	4
Mele,1994 16	Italy	ND	Related	Case-control	1,161	41.4	619	51	6
Nagata,1999 17	Japan	ND	ND	Case-control	109	48	25	55	4
Petro-Nustas, 2002 18	Jordan	ND	ND	Retrospective case-control	100	51.6	100	52.3	5
Zhang,2004 19	USA	White, black, others	ND	Population based case-control	519	61.2	449	61.4	7
Rauscher,2004 20	USA and UK	ND	ND	Cases and population-based controls in a US and Canadian case-control study	126	49.2	185	49.2	6
Andrew,2004 21	USA	White, non-white	ND	Case-control	156	58.6	69	59.4	5
Miligi,2005 22	Italy	ND	Irrelevant Related	Population-based case-control	NR	54.3	NHL, n=389; Hodgkin, n=77; MM, n=73; leukemia, n=140	NHL, n=58; leukemia, n= 57.6; MM, n=62.5; Hodgkin, n=41	7
Benavente,2005 23	Spain	ND	ND	Case-control	Hodgkin, n=187; leukemia, n=187	58	Hodgkin, n=179; leukemia, n=37	60	6
Heineman,2005 24	USA	White	ND	Population-based case-control	95	ND	112	ND	8
Tavani,2005^25^	Italy	ND	ND	Hospital-based case-control	233	ND	Hodgkin, n=23; NHL, n=89; MM, n=33; sarcoma, n=37	ND	7
De Sanjosé, 2006 26	6 European countries	ND	ND	Cases of lymphoid neoplasms hospital- or population-based controls	810	ND	780	ND	7
Lin,2006 27	USA	ND	ND	Large case-control study	82	ND	77	ND	7
Bluhm,2006 28	USA	Non-hispanic, white hispanic, white, black, others	ND	Hospital-based case-control	343	49.6	347	52.6	7
Chiu, 2007 29	USA	ND	ND	Population-based case-control	344	ND	49	ND	7
Morton,2007^30^	USA	ND	ND	Population-based multi-center study	408	65.8	509	65.6	9
Zhang,2008 31	USA	ND	ND	Population-based case-control	345	ND	185	21–74	6
Koutros,2009 32	USA	White	ND	Population-based case-control	500	ND	116	ND	6
Zhang,2009 33	USA	ND	ND	Population-based case-control	753	ND	680	ND	7
Sangrajrang, 2011 34	Thailand	ND	ND	Case-control study in the Thai population	122	ND	122	ND	8
Lv,2011^35^	China	ND	Irrelevant, related	Hospital-based case-control	NR	61	NR	59	5
Koutros,2011 36	USA and UK	White, hispanic, others	ND	Population-based case-control	235	65.12	159	65.84	8
Ros,2012^37^	The Netherlands	ND	ND	Population-based case- control	978	54.4	67	66.3	6
Fan,2012^38^	China	ND	Irrelevant	Hospital -based case- control	409	58.3	201	58.3	7
Guo,2014^39^	USA	Caucasian, African- American, others	ND	Population-based case-control	243	62.9	252	62.2	6

### Quality of studies

With the use of NOS as an assessment tool of case-control study quality, 16/31 studies were of good quality (13, 14, 22, 24–30, 33, 34, 36, 38, 40, 41), and 15/31 studies were of moderate quality (15–21, 23, 31, 32, 35–37, 39, 42, 43). A total of 28 studies between 1990 and 2015 were analyzed. The studies included subjects of different ethnicities and groups, although the majority of the studies had been conducted among Caucasians. Of the 31 studies, 5 were conducted in Asia (17, 18, 34, 35, 38), 7 inurope (16, 22, 23, 25, 26, 37, 40) 16 in the United States (13–15, 19, 21, 24, 27–33, 39, 41, 42), and 3 studies were jointly conducted in Europe and the United States (20, 36, 43).

**Fig 2:**
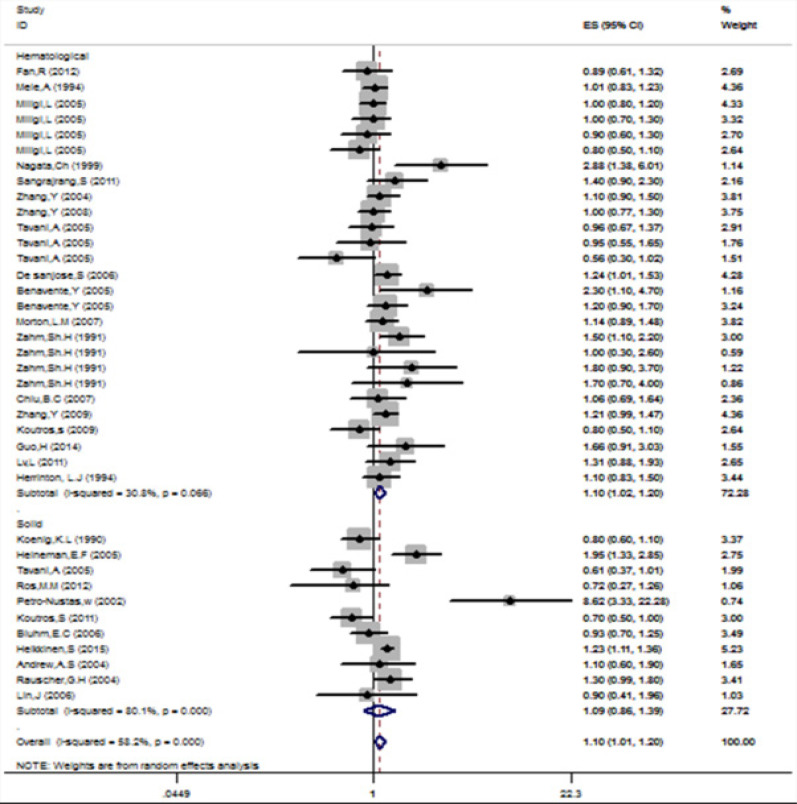
Forest plot of Risk of cancer to determination of relationship between ever use of hair dye and risk of cancer.

Analysis of results. The meta-analysis combined 28 studies (12,313 cases and 27,955 controls) to assess the association between hair dye use and cancer. It was demonstrated that the overall cancer risk was increased by 10% in the subjects compared with controls)OR=1.10; 95% CI: 1.01–1.21), and the heterogeneity between studies was moderate (I2=58.2%). There was no evidence of publication bias by funnel plot analysis (data not shown). In addition, subjects who regularly use hair dye had a 10% higher risk of hematopoietic system cancer compared with non-users)OR=1.10, 95% CI: 1.02–1.20) and the incidence of solid cancers was increased by 9% (OR=1.09, 95% CI: 0.86–1.39)(Fig. 2). There was no heterogeneity among studies on hematological cancers) I2=30.8% (, while there was significant heterogeneity among studies on solid cancers) I2= 80.1%.

**Fig 3:**
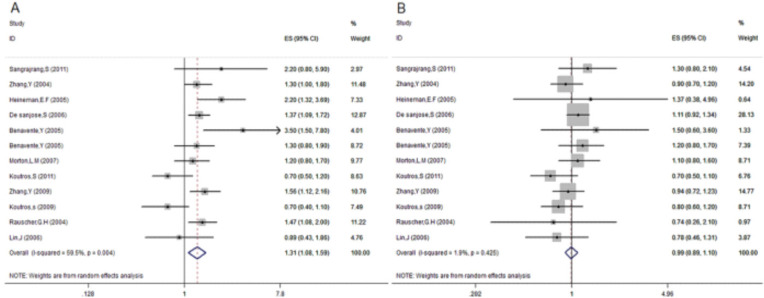
Association between production date of hair dye and risk of cancer. (A) Before 1980; (B) after 1980.

In addition, of the 28 studies included in the meta-analysis, 11 studies investigated hair dye manufactured before and after 1980 as a risk factor for cancer. In the present study, it was observed using hair dye manufactured before 1980 was associated with increased cancer rates by 31% compared with subjects who did not use hair dye) OR=1.31, 95% CI: 1.08–1.59) (Fig. 3A), with moderate heterogeneity across studies)I2= 59.5%(, while the use of hair dye manufactured after 1980 did not significantly affect the incidence of cancer (OR=0.99, 95% CI: 0.89–1.10 (Fig. 3B) and the heterogeneity across studies was very low (I2= 1.9%). There was no evidence of publication bias (data not shown).

**Fig 4:**
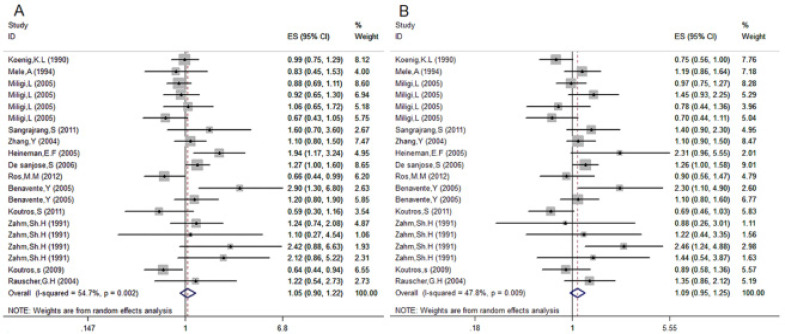
Association between color of hair dye used and risk of cancer. (A) Light hair dye; (B) dark hair dye.

According to the results of the pooled OR from the 13 studies that evaluated the effect of light and dark hair dye of cancer, the risk on cancer in women using dark hair dye increased by 9% in comparison with women who did not use hair dye (OR=1.09; 95% CI: 0.95–1.25) (Fig. 4A), and the heterogeneity between studies was moderate (I2=47.8%). There was no evidence of publication bias (data not shown). Women who used light hair dye had a 5% higher risk of developing cancer compared with those who did not use hair dye (OR=1.05, 95% CI: 0.90–1.22) (Fig. 4B), and the heterogeneity between studies was moderate (I2=54.7%). There was no evidence of publication bias (data not shown).

**Fig 5:**
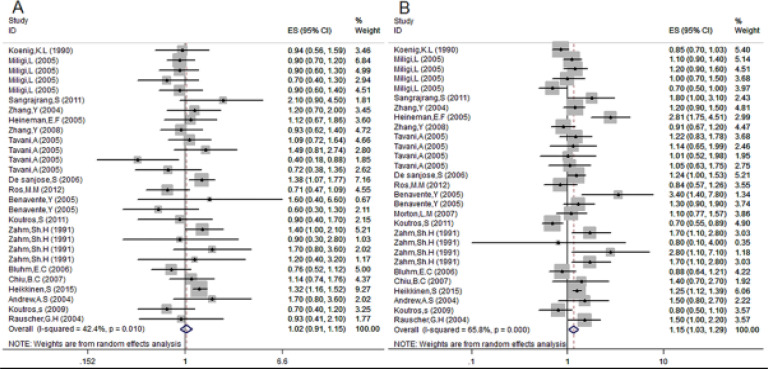
Association between type of hair dye used and risk of cancer. (A) Non-permanent; (B) permanent.

As indicated by the pooled OR from 18 studies that investigated the association of hair dye stability with cancer, subjects who used permanent hair dye had a 15% higher risk of developing cancer compared with those who did not use hair dye (OR=1.15; 95% CI: 1.03–1.29) (Fig. 5A, (and the heterogeneity between studies was moderate (I2=65.8%). There was no evidence of publication bias (data not shown). The use of non-permanent hair dye increased the risk of cancer by ∼2% compared with non-users (OR=1.02, 95% CI: 0.91–1.15) (Fig. 5B), and heterogeneity between studies was moderate (I2=42.4%). There was no evidence of publication bias (data not shown).

**Fig 6:**
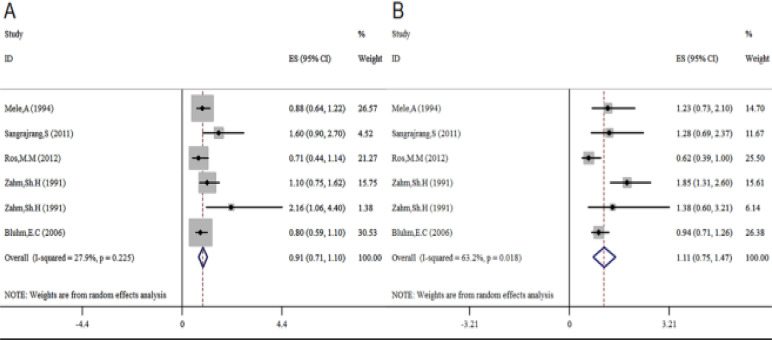
Association between duration of hair dye use and risk of cancer. (A) Less than 10 years; (B) over 10 years.

Based on the 5 studies that were conducted to investigate the role of duration of hair dye use as a risk factor for cancer, there was no difference in the risk of developing cancer between subjects who used hair dye for >10 years (Fig. 6A) and those who used hair dye for <10 years (Fig. 6B).

## Discussion

The present systematic review and meta-analysis evaluated 28 studies published between January 1990 and July 2017. The analysis revealed a 10% increased risk of cancer by ever use of any type of hair dye. Our results were according to production date (before or after 1980), duration of use, type of dye (permanent or non-permanent), and kind of color (light or dark).

Takkouche et al.^44^ reported a statistically significant increase (15%) in the risk of hematological malignancy with forever use of any type of hair dye (RR=1.15, 95% CI: 1.05–1.27). In a systematic-review and meta-analysis conducted by Gera et al.^45^ based on a case-control sur-vey, a statistically significantly increased risk of breast cancer was associated with forever use of any type of hair dye (RR=1.18, 95% CI; 1.03–1.36), whereas Towle et al.^46^ observed a statistically significantly increased risk of hematopoietic cancer (RR=1.29, 95% CI: 1.11–1.50), consistently with our results. When Takkouche et al.44 restricted the analysis to women, they observed no statistically significant increased risk (RR=1.04, 95% CI: 0.97–1.11).

In two meta-analysis studies conducted by Klesh et al. 2008 47 and Turati et al. 2013 48 that pooled the analyses based on case-control and cohort studies, no statistically significant association between ever use of any type of hair dye and solid cancer was observed (RR=1.01, 95% CI: 0.98–1.14 and RR=0.93, 95% CI: 0.82–1.05, respectively).

While another meta-analysis cancer related to ever use of any type of hair dye, Towel et al.^46^ conducted a systematic-review and meta-analysis based on pooled case-control studies, with results similar to our finding and a pooled RR of 1.09 (95% CI: 0.97–1.22); however, Takkouche et al.44 reported no statistically significant association of hair dye use with hematopoietic system cancers.

Comparison between light and dark hair dye revealed that the risk of cancer among dark hair dye users was 9% higher compared with that in the control group and that there was a significant association between light hair dye use and cancer. In a meta-analysis of case-control and cohort studies by Klesh et al.^47^, no association was observed between dark hair dye use and solid cancers (RR=0.94, 95% CI, 0.74–1.19), while another meta-analysis by Turati et al.^48^ reported an increased risk of solid tumors with the use of dark hair dye (RR=1.29, 95% CI: 0.98–1.71), following our results. Based on a review by Saitta et al in 2013 49, hair damage is accentuated with dark hair dye due to its higher precursor concentration; furthermore, the damaging properties of dark hair dye were proven by epidemiological evidence^5^, and there is a potential association with human malignancy.

These results indicate a significant association between cancer and the duration of hair dye use. The risk of cancer in subjects using hair dye for >10 years was 14% higher than that in the control group.

In two meta-analyses based on case-control studies by Klesh et al.^47^ and cohort survey by Turati et al.^48^, no statistically significant association was observed between duration of hair dye use for >20 years and solid cancers (RR=1.00, 95% CI: 0.85–1.19 and RR=1.01, 95% CI: 0.76–1.33, respectively).

In a systematic-review and meta-analysis^46^ based on 20 case-control and five cohort studies, there was a statistically significantly increased risk of hematological malignancies in subjects reporting use of hair dye for a duration of >15 years (RR=1.35, 95% CI: 1.13–1.62), which was following our results.

Nitrosamines, produced from secondary amines, are significant components of oxidative hair dyes^50^, and the carcinogenic properties and toxicity of nitrosamines were first highlighted by Magee and Barnes in 1956 51. Hair colorants contain ingredients with potential carcinogenic risks that demonstrate in genotoxicity studies and some animal experiments^52^. The European Commission Scientific Committee on Consumer Safety (SCCS) guidelines report that the minimum exposure time to nitrosamines to be considered a risk factor is 30 min; it may be inferred that continuous exposure to secondary amines for months or decades is associated with a higher risk^50^.

The strength of our analysis is the inclusion of all the studies from four databases examine the association between hair dyes and cancer, with a total of >12,313 cases, allowing us to investigate some of the critical variables in the assessment of exposure. However, there were certain limitations to the present study. A methodological limitation was the type of studies included in this evaluation. Selection bias is a particular problem inherent to case-control studies, leading to non-comparability between cases and controls. These studies are also prone to other forms of bias, such as recall and observer bias. If there is dissimilar reporting between cases and controls, the study would be prone to recall bias. If the cases were more likely to report hair dye exposure, the actual effect might be misestimated.

The present study aimed to conduct a systematic and meta-analytical assessment of the correlation between hair dye exposure and cancer. The results confirm this association, consistently with those reported by Gera et al.^45^, Towle et al.^46^, and Klesh et al.^47^. Since the SCCS of the European Commission recommended limiting the concentration of PPD as a carcinogenic chemical to a maximum o2%^53^, and The European Union Cosmetics Regulation allows a maximum of 6% PPD in hair dye products to prevent allergic reactions^54^, the consumers, while being aware of this potential association, are more likely to use products with an approved label based on standard confirmation.

## Abbreviations

MA: Mohadeseh Ahmadi
RAN: Reza Alizadeh-Navaei
AHO: Akbar Hedayatizadeh-Omran
ME: Mahboobeh Eslami
